# Mobile Application-Based Seoul National University Prostate Cancer Risk Calculator: Development, Validation, and Comparative Analysis with Two Western Risk Calculators in Korean Men

**DOI:** 10.1371/journal.pone.0094441

**Published:** 2014-04-07

**Authors:** Chang Wook Jeong, Sangchul Lee, Jin-Woo Jung, Byung Ki Lee, Seong Jin Jeong, Sung Kyu Hong, Seok-Soo Byun, Sang Eun Lee

**Affiliations:** 1 Department of Urology, Seoul National University Bundang Hospital, Seongnam, Korea; 2 Department of Urology, College of Medicine, Seoul National University, Seoul, Korea; University of L'Aquila, Italy

## Abstract

**Objectives:**

We developed a mobile application-based Seoul National University Prostate Cancer Risk Calculator (SNUPC-RC) that predicts the probability of prostate cancer (PC) at the initial prostate biopsy in a Korean cohort. Additionally, the application was validated and subjected to head-to-head comparisons with internet-based Western risk calculators in a validation cohort. Here, we describe its development and validation.

**Patients and Methods:**

As a retrospective study, consecutive men who underwent initial prostate biopsy with more than 12 cores at a tertiary center were included. In the development stage, 3,482 cases from May 2003 through November 2010 were analyzed. Clinical variables were evaluated, and the final prediction model was developed using the logistic regression model. In the validation stage, 1,112 cases from December 2010 through June 2012 were used. SNUPC-RC was compared with the European Randomized Study of Screening for PC Risk Calculator (ERSPC-RC) and the Prostate Cancer Prevention Trial Risk Calculator (PCPT-RC). The predictive accuracy was assessed using the area under the receiver operating characteristic curve (AUC). The clinical value was evaluated using decision curve analysis.

**Results:**

PC was diagnosed in 1,240 (35.6%) and 417 (37.5%) men in the development and validation cohorts, respectively. Age, prostate-specific antigen level, prostate size, and abnormality on digital rectal examination or transrectal ultrasonography were significant factors of PC and were included in the final model. The predictive accuracy in the development cohort was 0.786. In the validation cohort, AUC was significantly higher for the SNUPC-RC (0.811) than for ERSPC-RC (0.768, p<0.001) and PCPT-RC (0.704, p<0.001). Decision curve analysis also showed higher net benefits with SNUPC-RC than with the other calculators.

**Conclusions:**

SNUPC-RC has a higher predictive accuracy and clinical benefit than Western risk calculators. Furthermore, it is easy to use because it is available as a mobile application for smart devices.

## Introduction

Prostate cancer (PC) is the second most common cancer and is associated with the sixth highest cancer-related mortality in men worldwide [1]. Incidence rates of PC have increased in most countries, except in a few developed Western countries. There is a clear trend of rapidly increasing PC incidence in Asian countries, including South Korea [2], [3]. Thus, proper diagnosis of PC is a major problem in Asian countries. However, the use of transrectal ultrasonography (TRUS)-guided prostate needle biopsy (TRUS-Bx) to diagnose PC is accompanied by significant morbidity and mortality and is a considerable socio-economic burden [4]. For these reasons, the decision of whether to conduct a biopsy is of the utmost importance in actual practice [5].

**Figure 1:**
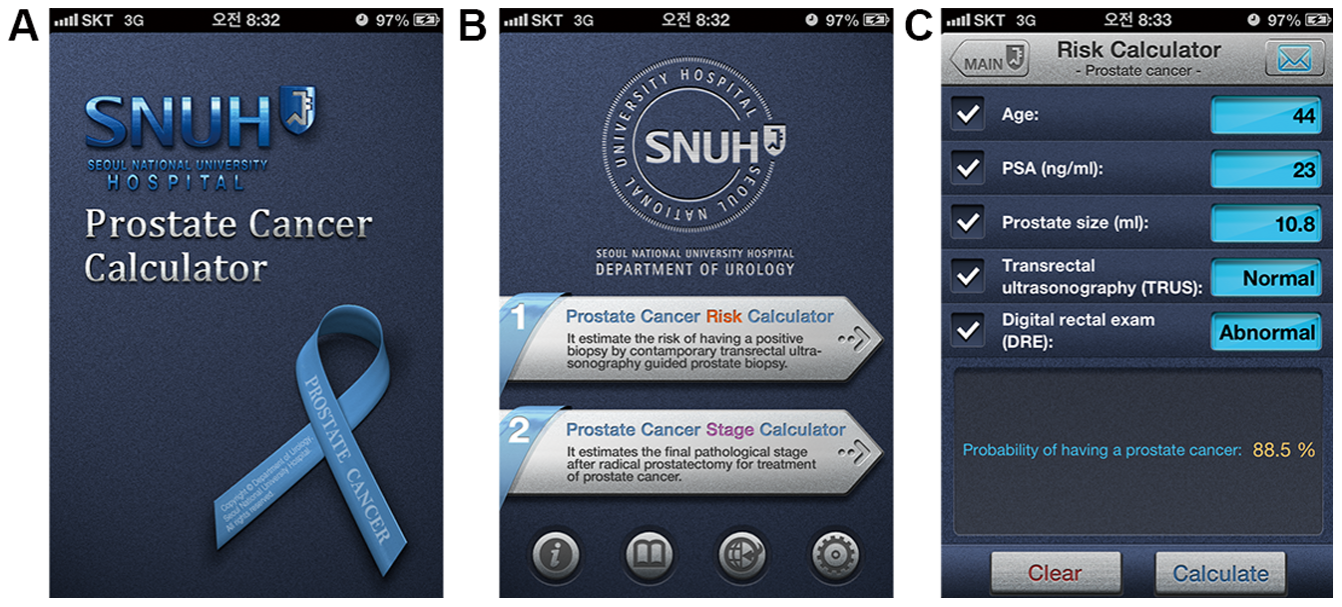
Seoul National University prostate cancer calculator (Version 1.1). (A) Main entry page of the calculator, (B) Selection page for “Prostate cancer risk calculator” or “Prostate cancer stage calculator”, and (C) Example of “Seoul National University prostate cancer risk calculator”.

To support this decision in various situations, many nomograms have been developed, primarily in the Western population [6]–[10]. To improve usability of these predictive tools, internet web-based probability calculators have been developed [11], [12]. These Western predictive tools are well validated and quite useful. However, the incidence and characteristics of PC in Asia are different from those in Western regions. Therefore, the generalized application of these Western tools in Asian men may require great precaution [13], [14]. Thus, we developed our own predictive model as a nomogram in 2011 using our patient cohort from 2003 through 2010. This nomogram predicts the probability of PC by the initial biopsy in Korean men. We then converted this nomogram into a mobile application, “Seoul National University Prostate Cancer Risk Calculator (SNUPC-RC)” in 2013. It has now been incorporated into the application, “Seoul National University Prostate Cancer Calculator” (Fig. 1). It can be operated either in Android or in iOS and is freely available on Google play store and the Apple App store. It was additionally validated in a recent cohort of our institution and was subjected to head-to-head comparisons with the representative internet web-based Western risk calculators which were developed in Western population. The compared risk calculators were the European Randomized Study of Screening for PC Risk Calculator (ERSPC-RC) and the Prostate Cancer Prevention Trial Risk Calculator (PCPT-RC) [11], [12]. Here, we describe its development and validation.

## Materials and Methods

### Ethics statement

The Institutional Review Board of Seoul National University Bundang Hospital (Seongnam, Republic of Korea) approved this study (approval number: B-1309/220-103). The need for informed consent from patients was waived by the Institutional Review Board because this was a retrospective analysis.

### Patient population and TRUS-Bx

This is a retrospective study which used the data collected from 5,278 consecutive patients who underwent TRUS-Bx at a tertiary referral center in South Korea. The study was conducted in 2 stages, with a time interval of 2 years. In the development stage, 3,924 cases from May 2003 through November 2010 were included. In the validation stage, 1,354 cases from December 2010 through June 2012 were included. We selected only men who underwent an initial biopsy in their life. The exclusion criteria were age less than 40 years, prostate-specific antigen (PSA) level more than 100 ng/ml, cases with missing data, and a biopsy core number of less than 12.

We only evaluated the contemporary systematic 12-core plus additional target TRUS-Bx, because this is regarded as the best practice and is widely accepted [15]. This biopsy scheme allows for maximal cancer detection and avoids repeat biopsy with adequate information. In our center, 2 experienced uroradiologists consistently performed TRUS-Bx with the systematic 12-core scheme using an 18-gauge needle. If TRUS indicated a suspicious lesion, 1 or 2 additional target biopsies per lesion were obtained. The prostate size was estimated using the prolate elliptical formula (height×width×length×π/6) in TRUS images [16]. Biopsy specimens were also consistently processed and examined by a single experienced uropathologist. Specimen examination and reporting were performed according to up-to-date consensuses and recommendations [17], [18].

### Development of the Seoul National University Prostate Cancer Risk Calculator

We performed the developmental study in 2011 using the aforementioned patient population. Of 3,924 cases reviewed, 3,638 men underwent an initial biopsy. Cases with an age less than 40 years (N = 62), PSA level more than 100 ng/ml (N = 72), biopsy core number less than 12 (N = 19), or missing data (N = 3) were excluded; thus, the “development cohort” consisted of 3,482 men.

Patients' age, PSA level, prostate size, palpable nodule by digital rectal examination (DRE), and suspicious lesions on TRUS were evaluated by logistic regression analyses [11], [19], [20]. In all analyses and models, the PSA level and prostate size were normalized by log-transformation. Significant variables detected by univariate analysis (p-value of <0.05) were included in the final multivariate model. The nomogram predicting the probability of PC was developed using this final multivariate logistic regression model. The predictive accuracy of this nomogram was evaluated by the area under the receiver operating characteristic curve (AUC). The agreement between the predicted probability and the actual outcome was evaluated by calibration plotting using 200 bootstrapping. In 2013, we converted this nomogram into a mobile application, “SNUPC-RC,” for iOS and Android systems, to improve usability.

### Validation and head-to-head comparison with ERSPC-RC and PCPT-RC

We conducted the validation study in 2013. Of 1,354 selected men, 1,161 underwent an initial biopsy. Cases with an age less than 40 years (N = 17), PSA level more than 100 ng/ml (N = 25), biopsy core number less than 12 (N = 2), or missing data (N = 2) were excluded, and 1,112 cases were finally analyzed as the “validation cohort.”

Head-to-head comparisons of SNUPC-RC with ERSPC-RC and PCPT-RC were conducted using the validation cohort. The individual probability of harboring PC was automatically calculated using the probability function of the model with the blinded data. The logit of ERSPC-RC is calculated as −2+1.1×log_2_ (PSA–2)−1.3×log_2_ (prostate size–5.4)+0.8×DRE+0.9×TRUS [11]. An abnormal DRE was assigned a value of 1, and an abnormal TRUS was assigned a value of 1; they were otherwise assigned a value of 0. The logit of PCPT-RC was calculated as −1.80+0.85×log_10_ (PSA)+0.27×family history+0.91×DRE−0.45×prior biopsy [12]. If there was a family history of PC or the DRE or prior biopsy was positive, the value of these parameters was 1, and otherwise, the value was 0. The probability function was calculated as exp (logit) / (1+ exp [logit]). The predictive accuracies measured by AUCs were compared using the DeLong method [21]. As a sub-population analysis, AUCs were also compared among an age group of 55–69 years (N = 553), because PSA screening is strongly recommended in this age group [22]. To test the clinical value of the predictive models, decision curve analyses (DCA) were conducted. The DCA visualizes the potential net benefit of the model at each threshold probability using a graph [23], [24].

The threshold probability of SNUPC-RC could be determined by each user. The diagnostic performances of SNUPC-RC with an exemplary threshold probability of 30% and a traditional PSA cut-off, >4 ng/ml were compared to demonstrate how many patients could avoid unnecessary TRUS-Bx [20], [25]. The diagnostic performances of ERSPC-RC and PCPT-RC were additionally calculated with the same threshold probability of 30%.

All statistical analyses were performed using R for Windows, version 3.0.1 (http://www.r-progect.org/), except the DeLong test. The DeLong test was performed using MedCalc, version 12.7.1.0 (MedCalc Software, Ostend, Belgium). A 2-sided p-value of <0.05 was considered statistically significant.

## Results

**Figure 2:**
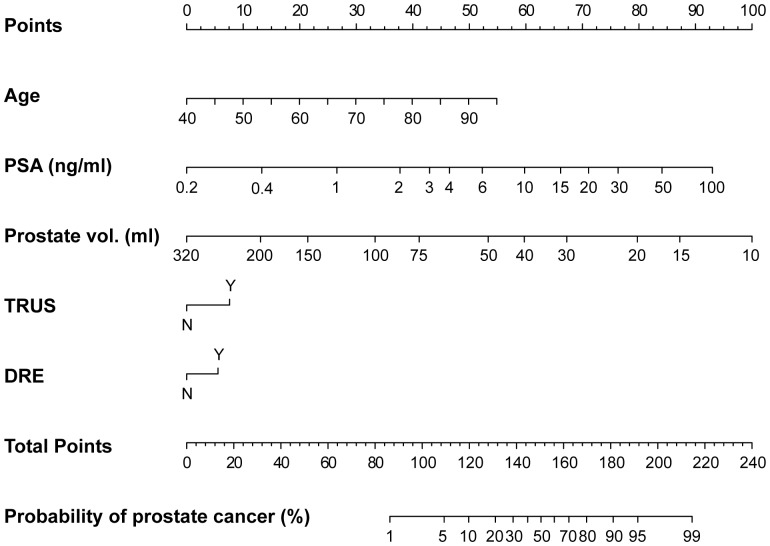
Seoul National University prostate cancer risk nomogram for prostate cancer probability prediction in Korean men.

**Figure 3:**
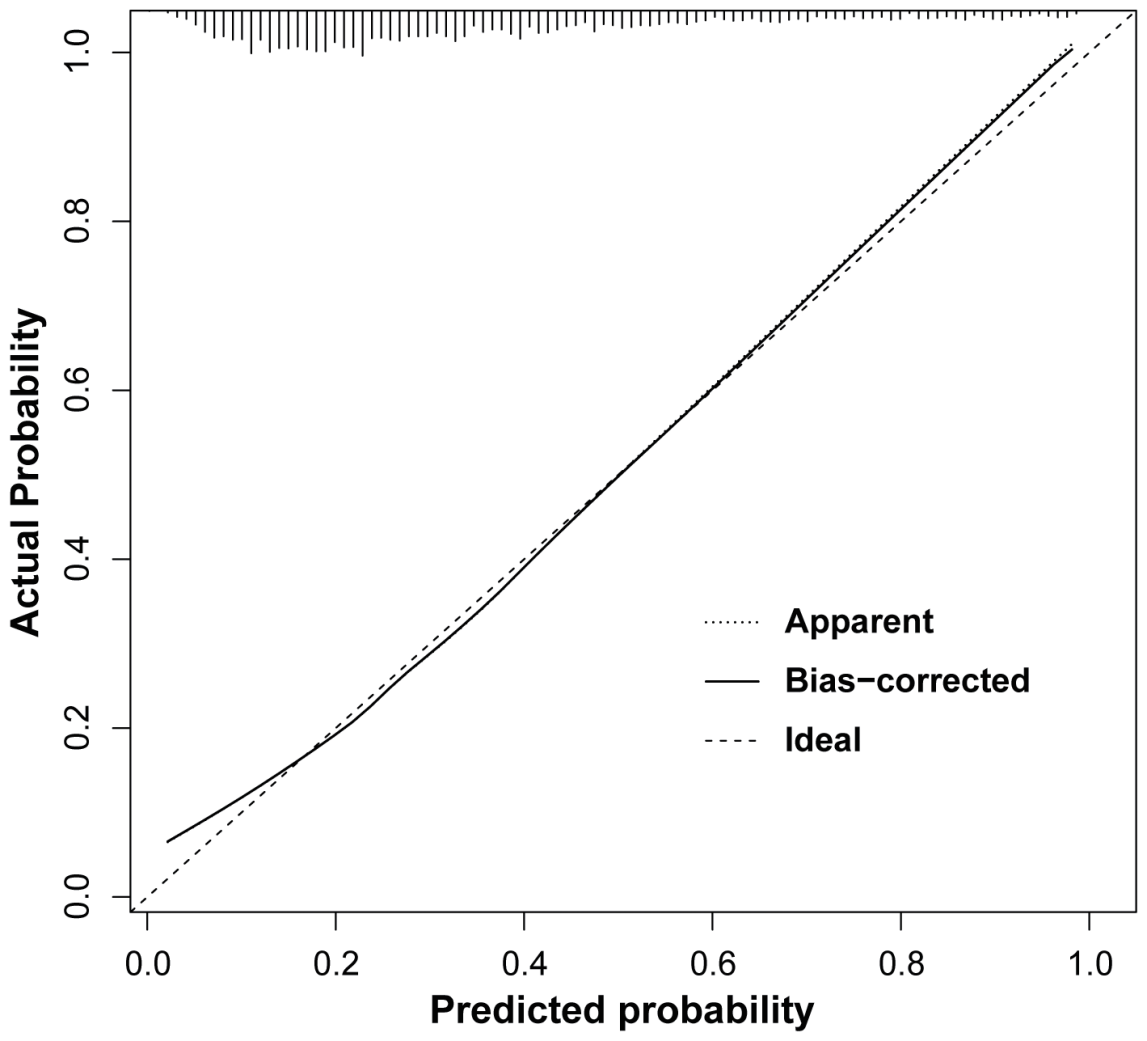
Calibration plot of the developed risk model.

The basic characteristics of the development and validation cohorts are summarized in Table 1. All evaluated variables were significantly associated with detection of PC in univariate as well as multivariate logistic regression analyses (Table 2). A graphical nomogram predicting the probability of PC in Korean men was constructed based on the final multivariate logistic regression model (Fig. 2). The predictive accuracy of this nomogram was 0.786 (95% CI, 0.779–0.802) calculated by AUC. The calibration plot demonstrated an almost perfect agreement between the predicted probability and the observed outcome fitted to the ideal line (mean absolute error 0.011) (Fig. 3).

**Table 1:** The basic characteristics of the development and validation cohorts.

	Development cohort	Validation cohort
Patients (n)	3,482	1,112
Age (years) (mean ± SD)	65.0±8.5	65.8±8.8
PSA (ng/ml) (mean ± SD)	9.8±11.5	10.7±13.0
Prostate size (ml) (mean ± SD)	46.6±23.2	42.1±19.9
Nodule by DRE (n) (%)	608 (17.5)	143 (12.8)
Abnormality by TRUS (n) (%)	875 (25.1)	216 (19.4)
Biopsy core number (n) (%)		
12	2,251 (64.6)	912 (82.0)
13	617 (17.7)	181 (16.3)
14	353 (10.1)	17 (1.5)
>14	261 (7.5)	2 (0.2)
Cancer detection (n) (%)	1,240 (35.6)	417 (37.5)

DRE: digital rectal examination, SD: standard deviation, TRUS: transrectal ultrasonography.

**Table 2:** Univariate logistic regression analyses and the final multivariate logistic regression model in the development cohort.

Variables	Univariate			Multivariate		
	OR	p	95% CI	OR	p	95% CI
Age	1.066	<0.001	1.056–1.076	1.074	<0.001	1.062–1.086
Log_10_ PSA	8.056	<0.001	6.329–10.254	10.792	<0.001	8.113–14.356
Log_10_ Prostate size	0.089	<0.001	0.058–0.135	0.008	<0.001	0.005–0.014
Abnormality by TRUS	2.329	<0.001	1.991–2.723	1.722	<0.001	1.434–2.069
Nodule by DRE	2.419	<0.001	2.025–2.889	1.487	<0.001	1.208–1.831
Intercept				1.233		

CI: confidence interval, DRE: digital rectal examination, OR: Odds ratio, TRUS: transrectal ultrasonography.

**Figure 4:**
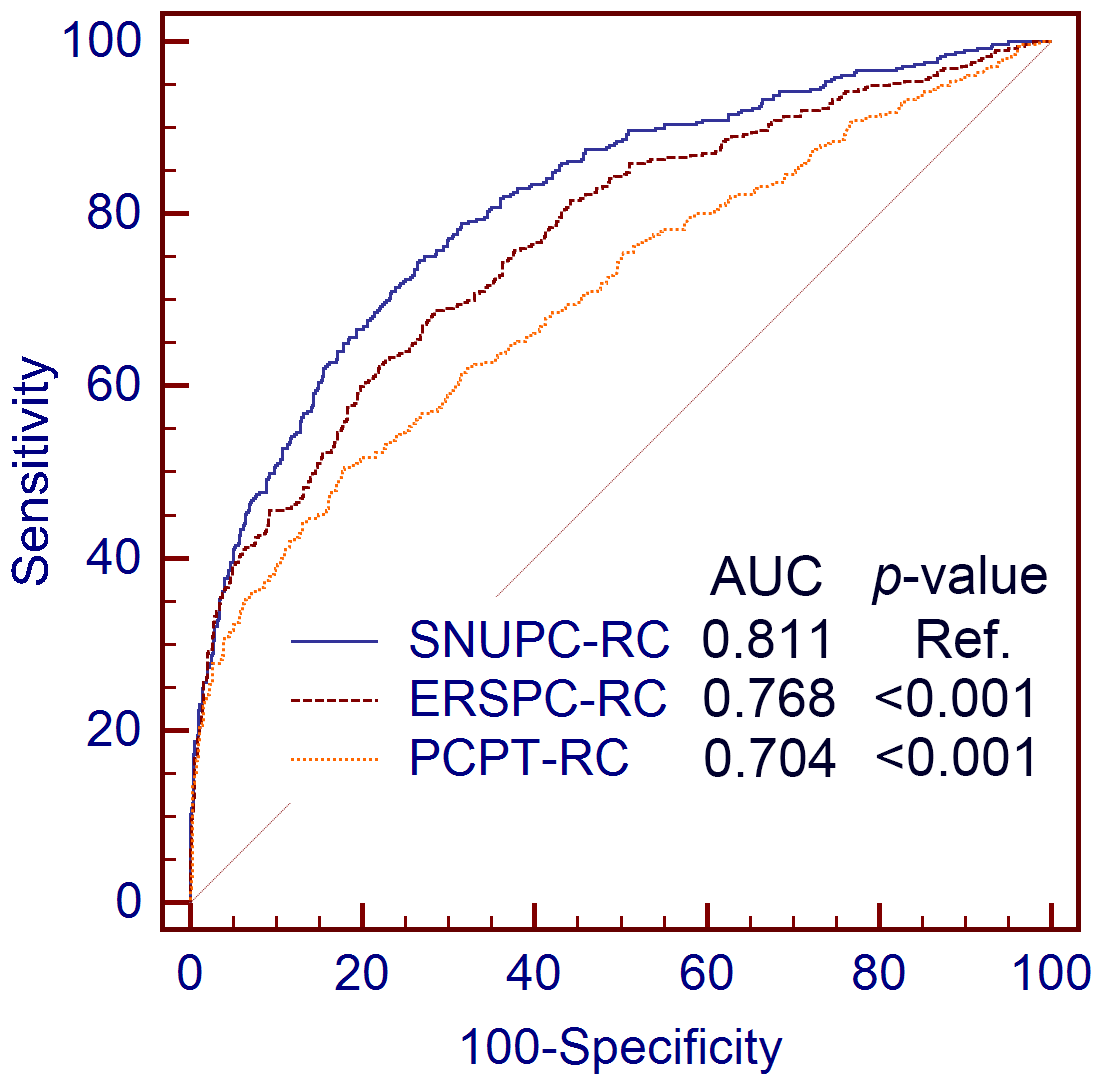
The receiver operating characteristic curves for the different calculators evaluated. Seoul National University prostate cancer risk calculator, European Randomized Study of Screening for Prostate Cancer Risk Calculator, and Prostate Cancer Prevention Trial Risk Calculator Were compared using the DeLong method.

**Figure 5:**
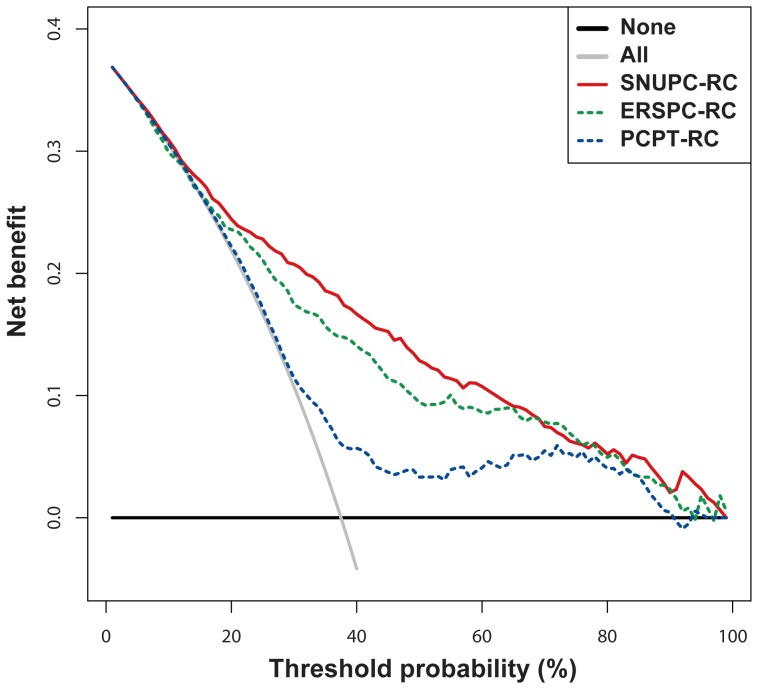
Decision curve analysis for the calculators evaluated. The Seoul National University prostate cancer risk calculator, European Randomized Study of Screening for Prostate Cancer Risk Calculator, and Prostate Cancer Prevention Trial Risk Calculator were analyzed.

In the validation cohort, the predictive accuracy of SNUPC-RC (AUC: 0.811; 95% CI, 0.786–0.833) was significantly higher than that of ERSPC-RC (AUC: 0.768; 95% CI, 0.742–0.792; p<0.001) and PCPT-RC (AUC: 0.704; 95% CI, 0.676–0.731; p<0.001) (Fig. 4). The clinical value of SNUPC-RC was also higher than that of ERSPC-RC and PCPT-RC (Fig. 5). SNUPC-RC had a higher net benefit than the other tools for almost all threshold probabilities (e.g. 0%–65%). Among an age group of 55–69 year, the predictive accuracy of SNUPC-RC (AUC: 0.785; 95% CI, 0.748–0.818) was also statistically higher than that of ERSPC-RC (AUC: 0.764; 95% CI, 0.726–0.799; p = 0.025) and PCPT-RC (AUC: 0.668; 95% CI, 0.627–0.707; p<0.001).

When using 4 ng/ml as the cut-off level of PSA, the sensitivity, specificity, positive predictive value, and negative predictive value were 86.8%, 26.9%, 41.6%, and 77.3%, respectively. If we set the threshold probability of SNUPC-RC as 30%, they were 76.3%, 61.3%, 56.3%, and 85.7%, respectively. By use of SNUPC-RC, an additional 239 men (21.5%) who did not have PC could avoid TRUS-Bx, whereas the number of undetected PC cases was only 16 (1.4%) when comparing PSA level of >4 ng/ml.

With the same threshold probability of 30%, the sensitivity, specificity, positive predictive value, and negative predictive value of ERSPC-RC were 71.7%, 64.8%, 55.0%, and 79.2%, respectively. PCPT-RC had 97.6%, 6.04%, 38.4%, and 80.8% in the same order.

## Discussion

PSA is an outstanding tumor marker among those for all malignancies. The PSA level is associated with the probability of PC diagnosis, prognosis, and treatment response [26]. To date, PSA represents the best surrogate marker for PC. Since the clinical application of PSA testing in the 1980s, the proportion of loco-regional PC has increased, whereas the incidence of metastatic disease has decreased [27]. This stage migration has also resulted in improvements in PC-specific survival over the past decades [1], [28]. Even though PSA is a good diagnostic marker for PC, the optimal cut-off value has not yet been established [20], [26], [29], [30]. This is due to suboptimal diagnostic performance. The positive predictive value of the PSA-based diagnosis in contemporary series is only 20–30% [20], [26], [29]. This means that approximately three-quarters of men undergo unnecessary TRUS-Bx, resulting in considerable social healthcare expenditure and unnecessary morbidity [4], [5]. In particular, increasing bacterial resistance to prophylaxis has resulted in substantial increases in the incidence of urinary tract infection and urosepsis [4], [31], [32]. In this situation, the best strategy might be simply limiting unnecessary TRUS-Bx, and when undergoing TRUS-Bx, the contemporary systematic 12-core biopsy method should be used to fully evaluate the risk of PC [5]. Therefore, we need a more comprehensive prediction model incorporating not only the PSA level but also other clinical parameters in the setting of the systematic 12-core TRUS-Bx. Then we can provide more precise data to assist in the process of tailored, shared decision-making with patients.

Many predictive models to diagnosis PC at initial biopsy have been made mostly with Western data [6], [8], [11], [12]. Even though these predictive tools have been validated in the Western population, their extrapolation to Asian, or more specifically to Korean patients should be done with caution [13], [14]. The predictive accuracies of ERSPC-RC and PCPT-RC in their original cohorts were 0.79 and 0.70, respectively. When applying these 2 risk calculators to the validation cohort consisting of Korean men, the accuracies were 0.768 and 0.704, respectively. In contrast, SNUPC-RC statistically outperformed ERSPC-RC and PCPT-RC with an accuracy of 0.811. The DCA also indicated a higher clinical benefit with SNUPC-RC than with ERSPC-RC and PCPT-RC. In the sub-population analysis among an age group of 55–69 years, AUC of SNUPC-RC was also statistically higher than those of ERSPC-RC and PCPT-RC. We selected this age group as this has shown the most benefit for PSA screening in the ERSPC trial [33]. The outperformance of SNUPC-RC may be caused by the different characteristics of the population and different practice patterns. The PSA screening rate in Korea is still lower than that in Western countries [2]. Furthermore, healthy Korean men have lower normal levels of PSA than their age-matched Western counterparts [34]. Another explanation is the difference in biopsy core number. In our cohort, we sampled 12 or more cores, whereas the ERSPC-RC and PCPT-RC are based on 6-core biopsies. Thus, the detection rates of these 2 Western series might be suboptimal. The variables in each risk calculator were somewhat different. We did not incorporate family history, because the incidence rate had been very low in Korea. We also left prior biopsy history out of SNUPC-RC, because the purpose of SNUPC-RC is to calculate the probability being diagnosed as PC by the initial biopsy not by repeat biopsy. In the context of clinical application, applying SNUPC-RC with a threshold probability of 30%, we could avoid unnecessary TRUS-Bx in approximately 20% of patients compared with PSA detection only. The diagnostic performance of SNUPC-RC was better than those of ERSPC-RC and PCPT with the same threshold probability of 30%. It could vary depending on the cut-off probability and shared decision-making process, we could always evade lots of unnecessary TRUS-Bx in reasonable range of threshold probability. We expect that we can provide better predictions and personalized shared decision-making for Korean men using SNUPC-RC. We will also be able to avoid unnecessary TRUS-Bx and reduce the socio-economic cost. Furthermore, SNUPC-RC has the potential to be used for other Asian populations after validation with their cohorts.

There were 1 Japanese nomogram and 1 Korean internet web-based risk calculator to predict the probability of PC in the Asian population; however, the numbers of biopsy cores used in developing these tools were 8 and 10, respectively. [35]. Western predictive tools are also seldom based on the contemporary 12-core biopsy [8], [11], [12], and their maximum core number was 10 [6]. However, a 12-core systematic biopsy incorporating apical and far lateral cores in the template distribution is strongly recommended. This biopsy methodology has been proven to result in maximal cancer detection, to eliminate the need for repeat biopsies, and to provide adequate information for developing a treatment plan [15]. Thus, previous predictive tools based on sampling of 6 to 10 cores might be too outdated to apply to present practice. It is worth mentioning that SNUPC-RC was developed by only incorporating men who had undergone a contemporary systematic 12-core TRUS-Bx. Therefore, SNUPC-RC can be used in contemporary practice.

Currently used clinical decision aids are risk groupings, decision trees, probability look-up tables, classification and regression trees, artificial neural networks, and nomograms. Among these, the nomogram is an excellent risk evaluation tool and has the highest discriminating power [13]. Visualization of the effect size of predictors to the risk might be an advantage of the nomogram. However, this requires a printout or screen shot of the nomogram. Furthermore, the exact number cannot be read from a nomogram. In comparison, internet web-based risk estimation tools provide exact probability and an easy user experience [14]. However, they require access to a device, such as a personal computer, with an internet connection, web browser, and hyperlink or typing of internet address. A mobile application-based risk assessment tool, such as SNUPC-RC, will be an alternative, because smart mobile devices are now being increasingly used. SNUPC-RC is freely available, and it can be used without internet access once downloaded. It is virtually ready to use anytime, anywhere once you downloaded it to your smart phone. You can counsel your patients even at bedside with your mobile smart devices. It is very user-friendly and provides enhanced smart functions. One example is that you can directly send and share the calculated results through an e-mail. To our knowledge, this is the first report regarding a mobile application-based cancer risk prediction tool in health care practice.

This study has many advantages. The risk model was developed and validated using large-scale cohorts that underwent the contemporary 12-core biopsy scheme, reflecting the current standard of care. Since the risk model was based on general clinical information, it could have a lower level of complexity. This report describes not only the development of SNUPC-RC, but also its additional validation and head-to-head comparison with Western internet web-based risk calculators over a long period. Clinical practice, TRUS-Bx, and pathologic examinations were consistently performed by experienced personnel according to up-to-date standards and recommendations. Furthermore, statistical analyses were performed at a high technical standard and were described in sufficient detail. We included the latest analytical techniques, such as DCA. We also adhered to standard guidelines in analyses and reporting of this field [36]–[38]. The current study also has several limitations. It depended on a retrospective methodological approach. Since our institution is a referral tertiary center, to use SNUPC-RC in a primary practice setting, it should be further validated in a primary practice cohort. Although SNUPC-RC better predicts the probability of PC, it does not establish the optimal cut-off level. Furthermore, it does not discern between clinically significant and insignificant PC. Finally, the real clinical impact after application of SNUPC-RC should be further evaluated over a long period.

In summary, this is the first report describing smart mobile application-based decision aids in cancer care. This application-based SNUPC-RC has a higher predictive accuracy than ERSPC-RC and PCPT-RC for estimating the risk of PC in the Korean population. Furthermore, SNUPC-RC has a higher clinical value than these 2 risk calculators. When using SNUPC-RC, a significant proportion of Korean men can avoid unnecessary TRUS-Bx, with only a small portion of undetected PC cases. It will provide clinically meaningful data for physicians and Korean patients during personalized shared decision-making for TRUS-Bx. When validated in other Asian countries, SNUPC-RC may also have the potential to be used for other Asian populations.
